# The neuroscience education readiness checklist for digital tools and methods

**DOI:** 10.3389/fncom.2026.1812915

**Published:** 2026-06-29

**Authors:** Mathew Abrams, Michael Denker, Damien Depannemaecker, Joao Alves Ferreira, Maren Frings, Meysam Hashemi, Jakob Kaiser, Judith Kathrein, Wouter Klijn, Trygve B. Leergaard, Milagros Marín, Karyn Onyeneho, Maja A. Puchades, Thomas Wachtler, Ekaterina Zossimova, Johannes Passecker

**Affiliations:** 1 INCF Secretariat, Karolinska Institutet, Stockholm, Sweden; 2 Institute for Advanced Simulation (IAS-6), Jülich Research Center, Jülich, Germany; 3 Aix-Marseille Universite, UMR INSERM 1106 Institut des Neurosciences des Systèmes, Marseille, France; 4 Institute of Pharmacology and Experimental Therapeutics, Faculty of Medicine, University of Coimbra, Coimbra, Portugal; 5 Coimbra Institute for Clinical and Biomedical Research (iCBR), Faculty of Medicine, University of Coimbra, Coimbra, Portugal; 6 Center for Innovative Biomedicine and Bio-Technology (CIBB), University of Coimbra, Coimbra, Portugal; 7 Forschungszentrum Jülich GmbH, Jülich Supercomputing Center (JSC), Simulation and Data Lab Neuroscience, Jülich, Germany; 8 Institute of Computer Engineering, Heidelberg University, Heidelberg, Germany; 9 Department of Teaching and Study Organization, Medical University of Innsbruck, Innsbruck, Austria; 10 Institute of Systems Neuroscience, Medical University of Innsbruck, Innsbruck, Austria; 11 Neural Systems Laboratory, Institute of Basic Medical Sciences, University of Oslo, Oslo, Norway; 12 DataJoint Inc., Houston, TX, United States; 13 National Institutes of Health, Bethesda, MD, United States; 14 Faculty of Biology, Ludwig-Maximilians-Universität München, Munich, Germany

**Keywords:** assessment, checklist, education, neuroscience, training resource

## Abstract

The integration of digital technologies in neuroscience, accompanied by a shift toward technology-enhanced learning environments, has created significant learning opportunities while simultaneously expanding the landscape of advanced training resources, ranging from biophysical simulations to high-dimensional neuroimaging and electrophysiological datasets. However, a critical “educator readiness gap” exists because the successful implementation of these tools often depends on the instructors' technical expertise and on their capacity to seamlessly integrate them into pedagogical practice. This paper introduces the Neuroscience Education Readiness Checklist (NERC), a 25-item self-assessment framework designed for creators and developers of neuroscience tools and training resources. NERC provides a structured approach that covers the major areas of concern in a comprehensive manner with actionable items. It organizes criteria into three overarching domains: general items and accessibility, instructor usability and workflow, and technical readiness. By addressing specific needs such as domain-specificity, pedagogical utility, and infrastructure requirements, NERC aims to reduce the burden on faculty members who evaluate training resource quality while providing important feedback for creators and developers. Developed through co-design workshops and refined with developer feedback, NERC provides a standardized approach to ensure neuroscience tools are not only technologically sound but also readily education-adoptable and scalable. While initial content has been curated through co-design, future validation will assess inter-rater reliability and predictive validity against adoption outcomes. Ultimately, NERC aims to foster a reproducible educational ecosystem, bridging the gap between innovative neuroscience and effective classroom implementation to ensure equitable access to high-quality neuroscience training.

## Introduction

Modern neuroscience education, particularly at secondary and tertiary levels, increasingly integrates digital technologies, e-learning, and self-regulated learning approaches (Geminiani et al., 2024; Chhetri, 2017; Nicolosi et al., 2018; Sandrone and Schneider, 2020). Within student-centered and competency-based learning paradigms, digital tools such as e-learning, gamification, and virtual reality have been shown to improve learner engagement, conceptual understanding, and retention (Aslan and Duruhan, 2021; Aytaç and Kula, 2022; Radianti et al., 2020; Dziuban et al., 2018). Advances in computing, data acquisition, and networked research infrastructures have made Digital Neuroscience Resources (DNRs) indispensable across all levels of neuroscience education, from the benchtop to the clinic. Examples of DNRs include brain atlases from Neurotorium's Atlas to the Allen Brain Atlas (Gilbert, 2018), digital cell atlases (Tecuatl et al., 2024) and interactive data analysis tools that are integrated into the EBRAINS research infrastructure. DNRs like Neuronify (Dragly et al., 2017) enable learners to explore intricate, multimodal neurobiological data that is impossible to capture in a static textbook format. This trend has been further accelerated by the COVID-19 pandemic, which triggered rapid shifts toward technology-enhanced learning environments (Almarzooq et al., 2020). Despite these advances, the rapid expansion of DNRs has outpaced the development of frameworks that support their effective adoption by educators.

Neuroscience education presents distinct pedagogical challenges due to the field's interdisciplinarity, and its use of complex, multiscale data (Akil et al., 2016). Training in neuroscience spans most of the major scientific disciplines including the fields of biology, psychology, physics, chemistry, medicine, mathematics, systems, data and computer sciences, engineering, artificial intelligence, robotics, as well as social sciences. Thus, neuroscience educators require specialist knowledge in multiple scientific disciplines to deliver high-quality learning experiences. Open-source DNRs developed by third parties can help to reduce the individual time burden associated with preparing teaching materials, allowing educators to focus on delivering engaging learning experiences. The development of DNRs is typically a community effort, driven by a team of experts from multiple adjacent disciplines.

DNRs such as interactive simulations, 3D brain visualizations, virtual dissections, computational modeling environments, and digital platforms for analyzing neuroimaging data can be used to supplement traditional teaching methods and facilitate independent learning (Gilbert, 2018; Ajani, 2024). They can provide more learner-centered and explorative real-world experiences, potentially improve knowledge acquisition and skills, enhance student engagement and interest in science, and address shortages of skilled professionals by strengthening educational capacity. Interactive viewer tools providing access to online atlas resources and collections of high-resolution histological images were not only important surrogates for physical dissection and microscopy courses during the COVID-19 pandemic but also paved the way for new approaches to interactive teaching of medicine and science. New DNRs have opened new possibilities for representing complex problems like dynamic neural processes in ways that are not possible through static textbooks. However, despite this potential, the increased availability of DNRs has not translated into consistent adoption in neuroscience education.

Computational neuroscience education is particularly challenging due to its inherently interdisciplinary nature, requiring learners to interpret biological problems and data, while simultaneously also developing technical competencies in mathematical modeling, programming and data analysis (Grisham et al., 2016). DNRs supporting this domain, from neural simulators such as NEURON, NEST, and Brian2 to data management platforms like DataJoint and standardized formats such as NWB, must scaffold this complexity while remaining accessible to researchers with heterogeneous technical backgrounds. While web-based interfaces and cloud-deployed workflows have begun lowering entry barriers (Spreizer et al., 2021), comprehensive frameworks for systematically evaluating the educational readiness of these diverse DNRs have been lacking. This gap leaves educators without structured guidance for selecting tools appropriate to their students' backgrounds and institutional constraints.

## The educator readiness gap: beyond tool functionality

Despite their technical sophistication, the successful implementation of DNRs is primarily determined by “educator readiness,” the capacity of instructors to seamlessly integrate these tools into existing pedagogical practices (Feng et al., 2025; Mekheimer, 2025). Many scientifically sound tools fail to achieve widespread adoption not because of technical flaws, but because they overlook the practical workflows and administrative constraints of the instructors responsible for their deployment (Amemasor et al., 2025).

There are several well-established evaluation frameworks: the Learning Object Review Instrument (LORI) (Nesbit and Li, 2004), the Mobile App Rating Scale (MARS) (Stoyanov et al., 2015), and instruments derived from the Technology Acceptance Model (TAM) (Davis, 1989; Venkatesh and Bala, 2008). These frameworks have made substantial contributions by providing structured, validated approaches to assess learning object quality, usability, engagement, and user acceptance across a wide range of educational and technological contexts. These general-purpose frameworks were not explicitly designed to address the educator adoption challenges and infrastructural constraints that increasingly shape the real-world implementation or the domain-specific requirements characteristic of basic and clinical neuroscience education. Here, we aim to bridge the educator readiness gap by providing practical, evidence-based guidance specifically for the developers of DNRs applicable for use in education.

## Purpose and scope: the neuroscience education readiness checklist (NERC)

Unlike evaluation frameworks primarily intended for *post-hoc* review, The Neuroscience Education Readiness Checklist (Table 1) is conceived as a self-assessment framework for developers and designers while also supporting informed adoption decisions by educators. By explicitly addressing data governance alongside educator workflows and technical constraints, NERC reflects the reality that neuroscience educators face where unclear data policies can prevent high quality tools from being adopted.

**Table 1:** The 25-item Neuroscience Education Readiness Checklist (NERC).

Item Nr.	Checklist criteria	Description	Self evaluation			
General items and accessibility						
1a	Usage	Number of students trained so far (estimate)				
1b	Language availability and requirement	In which languages is the DNR available? (e.g., English)				
1c	Associated cost estimate	What are the direct costs associated with the training resource for the trainer? (e.g., license cost)				
1d	Educational level	What is the expected education level target group? (Mark several if applicable)	□ Primary □ Secondary □ Undergraduate □ Graduate □ Post-Graduate			
1e	Coding competence level	Beginner (green)-Intermediate (yellow)-Professional (orange) (mark the lowest entry barrier)	NA			
1f	Self-study	What is the level of self (or group) study involved? < 33% (orange), Intermediate (yellow), >66% (green). (Mark the highest possible percentage)				
1g	Clear and measurable education use cases	Are the educational use cases for this training resource clearly described? Are they specific to neuroscience topics, measurable, student-centric? What are the Intended Learning Outcomes?	NA			
1h	Accessible multimedia	Visual information in multimedia such as videos or figures is described audibly or via text (captions/transcripts). All audio content includes transcripts.	NA			
14.8-7.4,-9.3498pt1i	Active engagement strategy	Does it utilize interactivity, relevant multimedia, real-world neuroscience examples, collaboration, choice, or appropriate gamification to maintain student engagement?	NA			
Instructor usability and workflow						
2a	Setup time and complexity	How long is the initial setup time for the instructor and the course? Is it straightforward and efficient?	NA			
2b	Classroom management features	Does it include relevant features for managing the online learning environment (e.g., communication, group management) if applicable?	NA			
2c	Clear and accessible documentation	Does it provide comprehensive, well-organized, and easily searchable user manuals, FAQs, and technical documentation for instructors?	NA			
2d	Availability of instructor packages	Does it come with existing free-to-use instructor packages, such as presentations, exercises or use cases that can be easily re-utilized in classrooms?	NA			
2e	Efficient student progress monitoring	Does the tool provide clear, actionable data/possibilities for instructors to easily track student engagement, progress, and performance?	NA			
2f	Facilitation of peer support/community	Does it provide mechanisms (e.g., forums) for instructors using the resource to connect and share practices?	NA			
2g	Instructor flexibility and customization options	Does it allow instructors some control to adapt the resource (e.g., select content, adjust settings, add context) to fit specific course needs and teaching styles?	NA			
14.7-7.4,0.1498pt2h	Guidance on assessment	Does it provide specific guidance, resources, or frameworks for effective assessment/grading?	NA			
Technical readiness						
3a	Installation requirements	Does the DNR need installation? If Yes, is it platform independent? Does it need soft-or specific hardware prerequisites? Does it require software dependencies prone to regular updates (high risk of issues)?	NA			
3b	Minimal and transparent technical requirements	What are the technical requirements? Does it minimize the need for specialized hardware/software or high-speed internet? Does it clearly communicate requirements? Does it consider equity? Does it avoid requiring users to download and install additional software or browser plug-ins where possible?	NA			
3c	Reliability and stability	Does the resource function consistently without frequent crashes, errors, or bugs on standard platforms/OS.	NA			
3d	Transparent and secure data privacy practices	Adheres to relevant privacy laws (FERPA, GDPR); clearly communicates data collection, usage, storage, security, and sharing policies. Minimizes PII collection. TrustEd Apps vetting is a plus.	NA			
3e	Clear data ownership and portability	Do policies clearly state ownership of data, reuse, and clarify ownership of user-generated content? Does the resource provide mechanisms for users to export/archive their data?	NA			
3f	Scalability	Can the infrastructure handle expected user loads (varying class sizes, concurrent use) without performance issues? What is the limit? (state number of users)	NA			
3g	Offline capability	Can core functionality operate without continuous internet access? Critical for field-based teaching or institutions with limited connectivity.		On-line	Partial	Full
3h	LTI/Learning tools interoperability conformance	Supports LTI standards.	NA	Yes/No		

The checklist aims to provide DNR developers with a self-assessment reflective opportunity to evaluate and improve their DNRs. At the same time, it will help educators with an improved overview of the capabilities and requirements of the DNR to assess suitability for their coursework. Many items follow a 3-tier color coding system. Orange indicates low/not met, yellow presents medium/partially met, and green indicates high/fully met; NA indicates non-applicability for a specific item. A DNR with predominantly green ratings and isolated yellow or orange items may be considered broadly ready for educational deployment, whereas a concentration of orange items within a single domain may indicate systemic barriers to adoption that should be addressed before classroom use. DNR developers are encouraged to prioritize orange-rated items, as these typically represent the most consequential barriers to effective use. Items marked NA should be excluded from the overall assessment profile rather than treated as deficiencies. A high proportion of NAs may suggest the checklist is being applied outside its intended scope. An annotated version of the checklist including guidance on the grading for each item is provided in the supplementary material.

The checklist was developed using a multi-stage, iterative co-design approach combining literature review, expert input, and developer feedback. An initial pool of checklist items was generated through a targeted review of literature on digital tool evaluation, educational technology frameworks, and usability standards (Davis, 1985; Venkatesh and Bala, 2008; Nesbit and Li, 2004; Tracey and John, 2007; Vargo et al., 2003; Gladman et al., 2020; Stoyanov et al., 2015; Trust, 2018; Watson and Anstey, 2018). Additional criteria were derived from the experiences of the INCF Training and Education Committee and the EBRAINS Education Task Force in providing training workshops to increase community adoption of the tools and services developed by the world's large-scale brain initiatives (e.g., US BRAIN, EBRAINS, CONP, Allen Institute), as well as the experience of the Neuromatch Academy. These sources informed a preliminary list of domains and candidate items. The preliminary checklist was refined through a series of co-design workshops and consensus-oriented meetings involving members of the EBRAINS 2.0 Education Task Force and the GA4GH Scientific Collaboration and Education Theme Team. Items were iteratively reviewed and refined through group discussion for 6 months. Selection was guided by predefined criteria, including: i) relevance to educator use, ii) clarity and interpretability, iii) feasibility of assessment, and iv) applicability across secondary and tertiary education contexts. Redundant or overly technical items were removed or merged. Consensus was reached through discussion. In a subsequent phase, the checklist was tested with a group of digital tool developers. Feedback focused on clarity, usability, and implementation feasibility, and informed further refinement of item wording and structure.

The scope of NERC is intentionally focused on criteria that directly impact an educator's ability to adopt, integrate, and manage DNRs effectively in their classrooms or labs. By addressing these criteria, tool creators can significantly enhance the likelihood of their DNRs being successfully implemented, thereby expanding the capacity for high-quality neuroscience education. The target audience of NERC primarily focuses on DNR developers to provide feedback and increase the DNR's value for educational purposes to serve educators and eventually students. By formalizing educator readiness as an explicit evaluation dimension, NERC contributes a novel perspective to the assessment of digital education resources. Rather than evaluating tools *post hoc*, it provides a formative, design-oriented framework that supports early identification of adoption barriers during tool development.

## General items and accessibility

The first domain of NERC (Table 1) addresses the foundational logistics and accessibility of a DNR, ensuring it can reach a diverse student population regardless of their background or resources. The rationale here is rooted in the equity of access. High costs and specific hardware requirements (Item 1c) are primary drivers of educational inequality, as institutions in emerging economies or resource-constrained settings often lack the capital to maintain expensive licensing or specialized equipment (Barikzai et al., 2024; Toofaninejad et al., 2025; Antoninis et al., 2023). By evaluating items such as Language Availability (1b), Coding Competence (1e), and Accessible Multimedia (1h), NERC prompts developers to consider Universal Design for Learning principles (Rose, 2001). For example, in neuroscience, where complex concepts often rely on high-fidelity visualizations as part of presentations, providing accurate captions and transcripts (1h) is essential for students with different learning needs or those in non-English speaking environments. This section ensures that a DNR's “Educational Level” (1d) is transparently aligned with its target group, preventing the common educational readiness gap where advanced research tools are deployed to undergraduates without sufficient context-based scaffolding.

## Instructor usability and workflow

The second domain focuses on the practical integration of DNRs into the teaching workflow, directly addressing the dual assessment burden. Literature suggests that even sophisticated DNRs fail if they neglect the practical workflows of instructors (Dinçer, 2024; Casimo, 2023). The rationale for including Setup Time (2a), Classroom Management Features (2b), and Instructor Packages (2d) is to lower the entry barrier for faculty who must manage limited time resources (Bond et al., 2020). By assessing Student Progress Monitoring (2e) and Guidance on Assessment (2h), NERC ensures that the tool supports the instructor's need to evaluate both the DNR's suitability and the students' actual learning outcomes. This moves the evaluation beyond engagement toward pedagogical utility, ensuring that DNRs facilitate, rather than hinder, the delivery of neuroscience content.

## Technical readiness and infrastructure

The final domain provides a check of the DNR's technical stability and legal data practices, which are critical for institutional adoption. The rationale stems from the fact that infrastructure limitations, such as a requirement for costly GPU capabilities or specific local hardware (3b), often implicitly impede the scalability of DNRs; studies show that moving toward cloud-deployed workflows and browser-based interfaces is essential for breaking these accessibility barriers in the classroom (Jalili et al., 2020; Spreizer et al., 2021; Delogu et al., 2025). This section evaluates Platform Compatibility (3a) and Reliability (3c) to ensure the DNR functions consistently across diverse educational environments without frequent bugs or crashes. The technical vetting items are essential for developers to create reproducible and accessible training ecosystems that allow students and educators to focus on scientific use cases rather than troubleshooting software dependencies.

## Examples for the application of NERC

In the following section we provide an overview of five DNR examples via a perspective application of NERC. Filled-out NERC checklist assessments for all five examples are found in the Supplementary Tables 2–6.

## Example 1: advanced computational modeling and inference training module

The Training Module developed as part of a Computational Neuroscience summer school is based on a curriculum that is structured in a manner that provides learners with foundational computational modeling knowledge to full inference pipelines (e.g., Markov chain Monte Carlo) (Ziaeemehr et al., 2025; Baldy et al., 2025). Application of NERC to the advanced computational modeling and inference training module revealed a high level of readiness across pedagogical, instructor workflow, and technical governance dimensions. The module demonstrates strong coherence between learning objectives and instructional design, with educational use cases (1g) explicitly targeted toward advanced undergraduate and graduate learners. Learner-centered learning is supported with interactive Jupyter notebooks that enable learners to actively explore model parameters and inference which enables self-study (1f). This aligns with NERC criteria for clear educational use cases, active engagement strategies, and targeting of appropriate educational levels.

The evaluation of Instructor Usability and Workflow (Domain 2) indicates that the module successfully mitigates classroom adoption barriers despite its underlying technical complexity. Integration within the EBRAINS Research Infrastructure reduces setup time (2a) by removing dependencies on local software installation. However, account creation requirements increase barriers and impact item 2a. Scalability is, however, evidenced by successful deployment in multiple training contexts, including international summer schools and mentored research programs such as the Google Summer of Code. Instructors are supported by comprehensive documentation and reproducible workflows, which facilitate the assessment of student performance based on both code execution and the interpretation of inference results. Furthermore, the module aligns with institutional requirements for Technical Readiness (Domain 3). The use of shared, version-controlled datasets and workflows supports reproducibility and data portability, and adherence to EBRAINS data governance policies ensures compliance with relevant legal and ethical standards (3d, 3e). This case illustrates how high technical complexity does not preclude educational readiness when instructor workflows, documentation, and data governance are explicitly addressed.

## Example 2: NIH toolbox: standardized neurobehavioral assessment platform

The NERC assessment of the NIH Toolbox for Assessment of Neurological and Behavioral Function yielded a mixed readiness profile, characterized by high scientific validity alongside notable barriers in instructor workflow and assessment integration (Shields et al., 2020; Fox et al., 2022). The profile was characterized by strong pedagogical validity and technical robustness, but notably weaker in terms of instructor workflow and assessment integration. In terms of pedagogical alignment, the NIH Toolbox offers a clearly defined and well-validated educational use-case appropriate for undergraduate, graduate, and clinical neuroscience training. The standardized assessment battery provides learners with exposure to the core concepts of cognitive, sensory, motor, and emotional function, supported by normative datasets and reproducible scoring procedures. These features align strongly with NERC criteria regarding clear and measurable educational use cases (1g) and appropriate targeting of educational level (1d). However, the rigidity inherent to standardized testing protocols limits opportunities for active experimentation or inquiry-driven learning, which reduces its alignment with NERC criteria for active engagement strategies (1i) and flexible self-study pathways (1f).

Evaluation of Instructor Usability and Workflow (Domain 2) highlighted substantial adoption barriers. While the platform is scientifically rigorous, its deployment places demands on instructors, including the need to interpret outputs for instructional use and manage technical integration steps. These challenges are reflected in limited availability of instructor-ready materials (2d) and constrained assessment guidance (2h). Consequently, instructors must translate data outputs into course-specific learning objectives, which may reduce usability in time-constrained environments, despite the platform's scientific value. Conversely, the platform demonstrates strength in Technical Readiness (Domain 3), particularly in adherence to established data privacy standards (3d) and a clear governance framework for data handling and ownership (3e). The system is stable and scalable for research environments (3c, 3f), supporting broad deployment. However, platform dependence on iPhone Operating System (or iOS) infrastructure and required hardware introduces accessibility and equity considerations (3a, 3b) that may limit deployment in resource-constrained institutions. Overall, the NIH Toolbox illustrates a recurring pattern in neuroscience education tools: high scientific rigor and research utility do not always translate into seamless integration into instructional workflows due to instructor burden and implementation constraints.

## Example 3: meshview - 3D atlas viewer

MeshView is a web-based rodent 3D atlas viewer for real-time display of surface mesh data representing structural parcellations from volumetric atlases, such as the Waxholm Space Atlas of the Sprague Dawley Rat Brain [MeshView RRID: RRID:SCR_017222;(Puchades et al., 2025)]. The DNR facilitates the exploration and analysis of mouse and rat brain anatomy by allowing users to toggle the opacity of specific parcellations or apply arbitrary cross-sections to examine the spatial relationships of internal structures, such as the characteristic morphology of the hippocampus. This DNR is integrated in different analytical workflows available on the EBRAINS research infrastructure like the QUINT online or LocaliView workflows (https://quint-online.apps.ebrains.eu/ and https://localiview.apps.ebrains.eu/).

This example illustrates the educational readiness of a typical neuroscience software developed for research purposes. The mapping showed that while MeshView scores high in Technical Readiness (Domain 3) and General Accessibility (Domain 1) due to its browser-based, device-agnostic nature, it lacks essential features in Instructor Usability and Workflow (Domain 2). Specifically, the tool lacks integrated measurable education use cases (1g) and instructor packages (2d). For effective classroom adoption, the evaluation identified a need for dedicated video tutorials and built-in mechanisms for student progress monitoring and assessment (2e, 2h). This example highlights how NERC can serve as a roadmap for research developers to transition highly functional scientific tools into viable educational resources.

## Example 4: elephant analysis toolkit

The Electrophysiology Analysis Toolkit (Elephant; RRID:SCR_003833) is a Python based open-source library to perform analysis of experimental and simulated brain activity data on the level of networks of spiking neurons and electrophysiolgical population signals (Denker et al., 2018; Senk et al., 2017). A NERC self-assessment reveals that the financial and technical barriers to gaining access to Elephant are low, as the library is openly available under the BSD license, can be installed locally via the standard package manager of Python (pip), is part of the EBRAINS software distribution (ESD) and is pre-installed as part of multiple open online services, such as the interactive Jupyter Lab programming environments of EBRAINS or OpenSourceBrain (Gleeson et al., 2019). Regarding Technical Readiness (Domain 3), the hardware requirements are minimal, and extensive unit testing ensures operational stability. Nevertheless, as a software programming library, Elephant faces the challenge of a comparatively higher barrier to entry as compared to graphical user-facing DNRs because a rudimentary knowledge of programming in the Python programming language is required for exploration. This leads to a more advanced student target group, starting at undergraduate levels (1d, 1e). NERC inspired the idea to develop specific training materials for novice proficiency groups that abstract coding complexity to focus on the underlying neural data science.

While Elephant's Jupyter-based exercise materials have successfully supported in-person, online, and inverted classroom scenarios over the past decade, NERC item 2d (Instructor Packages) identified a gap: the lack of standardized documentation for instructors to automate the deployment of customized course materials. Furthermore, the assessment of General Items and Accessibility (Domain 1) highlighted that while interactive teaching content is abundant (https://tutorials.python-elephant.org), it is not integrated into an overarching course module. To consolidate these resources, future iterations will focus on explicitly outlining measurable education use cases (1g) and integrating the existing multimedia content to enhance learner engagement (1h, 1i). Taken together, the example assessment demonstrates how the NERC self-assessment can assist in pinpointing very concrete actions to improve the usability of library documentation for teaching scenarios.

## Example 5: brainscales-2: neuromorphic system

BrainScaleS-2 is a neuromorphic system that emulates the behavior of individual spiking neurons as well as networks of neurons. Unlike conventional simulations, which solve differential equations numerically, BrainScaleS-2 is based on physical electronic circuits that directly mimic the dynamics of neurons and synapses. Access to the system is facilitated through interactive Python-based Jupyter notebooks utilizing high-level modeling APIs (Müller et al., 2022). These resources support a spectrum of neuroscientific inquiry to explore both neuroscientific concepts, event-based computation, and machine-learning-inspired training. Introductory notebooks use interactive widgets to provide a low entry barrier (Domain 1), while advanced notebooks focus on programming tasks. Several notebooks are specifically designed for secondary school students and introduce neuroscientific concepts using clear and accessible language. More advanced notebooks are actively used in an undergraduate physics laboratory course at Heidelberg University and are regularly presented at neuroscience and neuromorphic computing conferences throughout the year. To ensure Accessibility (Domain 1), resources are hosted on GitHub and support machine translation to facilitate adoption across diverse linguistic contexts (currently available in English, and partly in German). Because BrainScaleS-2 emulates neuronal and synaptic behavior using dedicated physical neuromorphic hardware, access to this hardware is required. To ensure Technical Readiness (Domain3), the systems and software were integrated into the EBRAINS Research Infrastructure (and its EBRAINS software distribution). Students and teachers can register with EBRAINS free of charge, and guest accounts can be requested to facilitate straightforward use of the system in classroom settings. However, the NERC evaluation identified potential for optimization in Instructor Usability and Workflow (Domain 2). An EBRAINS user account (or EBRAINS guest accounts) is needed to access the hardware system. After that, the resources can be deployed with a “single-click” minimizing Setup Time and Complexity (2a). Furthermore, as these DNRs were originally designed for conference tutorials, they currently lack dedicated classroom management features (2b) and structured guidance on assessment (2h). Future refinements should incorporate questions and tasks to assess student progress. Furthermore, external pedagogical libraries could be integrated to support progress monitoring and the articulation of more granular, measurable educational use cases.

The five illustrative cases presented span computational modeling modules, standardized neurobehavioral platforms, anatomical viewers, neuromorphic hardware, and software libraries collectively validate NERC as a comprehensive framework for identifying and mitigating adoption barriers. The qualitative profiles derived from these five assessments are summarized in Supplementary Table 1, providing a visual comparison of educational readiness across different classes of DNRs. These patterns align with existing adoption data: resources that achieved higher readiness scores in the Instructor Usability and Technical Readiness domains show more consistent evidence of successful, sustained classroom use. In contrast, the “Orange” barriers identified for the NIH Toolbox and MeshView highlight specific gaps in instructor-facing support and assessment integration that frequently impede the transition from research-grade software to education-ready tools. These cases demonstrate that NERC effectively differentiates between technical maturity and practical educational adoptability. For instance, the contrast between the Advanced Computational Modeling module and the NIH Toolbox underscores that scientific rigor and technical stability do not inherently translate to classroom readiness without an integrated approach that prioritizes instructor workflows and pedagogical scaffolding. In the case of the NIH Toolbox, the lack of instructor-facing support highlights how scientific value can be overshadowed by the practical burdens of deployment in time-limited teaching environments. For research-grade DNRs such as MeshView and Elephant, the NERC assessment provided a structured diagnostic that pinpointed specific deficiencies in documentation and assessment features, illustrating the framework's role as a formative self-assessment tool that provides developers with concrete, actionable steps to improve the educational readiness of their DNR. Furthermore, the BrainScaleS-2 example highlights the framework's ability to navigate specialized hardware requirements while surfacing potential barriers related to setup complexity and equitable access. By making adoption-critical factors explicit (like data governance, setup time, and licensing costs), NERC surfaces constraints that often remain implicit in traditional technical or pedagogical evaluations.

## Discussion

This study introduces the Neuroscience Education Readiness Checklist (NERC), a structured framework for evaluating the educational adoptability of DNRs. NERC comprises 25 items categorized into three domains: general items and accessibility, instructor usability and workflow, and technical readiness. It is specifically tailored to address the unique requirements of neuroscience and its adjacent educational disciplines. NERC is designed as a formative reflection tool rather than a summative certification or accreditation instrument. This qualitative approach intentionally avoids rigid scoring or arbitrary weighting, instead providing a diagnostic profile that highlights specific areas requiring developer attention to improve a resource's educational adoptability. The checklist is most effective when applied iteratively throughout the development lifecycle. Developers are encouraged to perform an initial assessment to identify “Orange” barriers which represent the most consequential bottlenecks. The DNR should subsequently be re-evaluated after implementing refinements to track longitudinal progress in readiness. Given that the readiness of any digital tool for educational implementation depends on a complex and interdependent array of factors (Mosa et al., 2016; Feng et al., 2025), no single general assessment framework can realistically capture all relevant dimensions. As a result, a unified assessment standard has yet to emerge. Existing frameworks like LORI, MARS, MARul, and TAM (Davis, 1985; Venkatesh and Bala, 2008; Nesbit and Li, 2004; Tracey and John, 2007; Vargo et al., 2003; Gladman et al., 2020; Stoyanov et al., 2015) omit educator constraints and often lack domain-specificity required for rigorous assessment within specialized research-driven or medical contexts. Furthermore, as digital educational resources are increasingly deployed within blended and hybrid learning environments, their evaluation must extend beyond content quality to include their capacity for flexible delivery and support for collaborative learning.

The illustrative use cases presented here demonstrate how NERC surfaces adoption-relevant constraints that are not always apparent when evaluating DNRs based solely on pedagogical quality or user engagement. To further contextualize this contribution, it is important to position NERC relative to existing frameworks commonly used to assess digital learning resources and educational technologies. As summarized in Table 2, NERC differs from these frameworks in both scope and intended use. LORI emphasizes the pedagogical quality of learning objects (e.g., alignment with learning goals, feedback, motivation, and presentation design), but places comparatively less emphasis on instructor workflow, technical deployment, or institutional infrastructure constraints. MARS provides a robust and widely adopted instrument for rating mobile applications that focuses on engagement, functionality, aesthetics, and information quality; but it remains largely agnostic to curricular integration, assessment practices, and classroom-scale deployment. TAM-based instruments, while extensively used to explain and predict technology adoption through constructs such as perceived usefulness and perceived ease of use, are primarily explanatory rather than prescriptive since they often identify attitudes and intentions but offer limited guidance on concrete design or infrastructural changes required to improve educational readiness.

**Table 2:** Comparison of NERC against other technology evaluations frameworks.

Dimension	NERC (Neuroscience Education Readiness Checklist)	LORI (Learning Object Review Instrument) (Vargo et al., 2003)	MARS (Mobile App Rating Scale) (Stoyanov et al., 2015)	TAM/TAM-based instruments (Davis, 1985; Venkatesh and Bala, 2008)
Primary purpose	Assess educator readiness and adoption feasibility of neuroscience education resources	Evaluate pedagogical quality of learning objects	Assess quality of mobile apps (engagement, functionality, aesthetics)	Explain and predict technology acceptance
Typical users	Developers, instructional designers, evaluators	Educators, instructional designers, reviewers	Researchers, app evaluators	Researchers, institutions
Domain specificity	High (neuroscience-specific)	Low (domain-agnostic)	Low (domain-agnostic)	Low (domain-agnostic)
Instructor workflow focus	High (setup time, instructor packages, assessment integration)	Moderate	Low	Indirect
Technical readiness and infrastructure	High (installation, scalability, compatibility, data governance)	Low–Moderate	Moderate	Low
Accessibility and equity focus	Explicit	Variable	Limited	Not explicit
Assessment and learning outcomes support	Explicit	Explicit	Indirect	Not inherent
Data privacy and governance	Explicit (GDPR, FERPA, portability)	Rarely addressed	Partially addressed	Not addressed
Scoring approach	Checklist with tiered readiness levels	Likert-scale ratings per dimension	Likert-scale ratings	Likert-scale constructs
Role relative to NERC	Not applicable	Evaluates pedagogical quality	Evaluates app quality	Explains acceptance behavior
Value add of NERC	Operational educator readiness, technical governance, neuroscience constraints	Adds instructor deployment, infrastructure, and policy aspects	Adds educator adoption, curricular fit, and assessment alignment	Adds objective “readiness”

NERC complements established frameworks by operationalizing educator readiness as a pivotal evaluation dimension. While existing tools like TAM or LORI focus on user acceptance or specific pedagogical quality, NERC addresses the operational conditions, such as data governance, infrastructure scalability, and assessment integration, that frequently dictate whether a DNR is sustained or abandoned within institutional constraints. In this regard, NERC functions as an actionable bridge between tool development and classroom implementation.

NERC's second domain directly mitigates the dual assessment burden faced by faculty, who must often evaluate a tool's quality while simultaneously using it to measure student progress. By providing structured Guidance on Assessment (item 2h) and Instructor Packages (2d), the framework allows educators to efficiently decouple surface-level engagement from the attainment of objective learning outcomes within limited time resources.

As AI-assisted features become increasingly integrated into DNRs, their implications for educational readiness require careful consideration. Large language models (LLMs) are now being embedded in computational platforms to support code generation, data interpretation, and troubleshooting–capabilities that can lower the entry barrier for learners with limited programming experience (Kasneci et al., 2023). For instance, AI-powered assistants such as GitHub Copilot, now integrated into Jupyter-based environments commonly used in neuroscience workflows, can provide real-time code suggestions and debugging support during data analysis exercises, potentially reducing instructor support burden while scaffolding learner autonomy. Beyond coding support, recent developments in open neuroscience infrastructure illustrate broader AI integration: the DANDI Archive, which hosts neurophysiology data in NWB format, now offers an LLM-powered exploration assistant that enables users to query, visualize, and analyze datasets through natural language interaction, as well as an automated generator for dataset-specific instructional materials (Magland et al., 2025). Similarly, adaptive learning systems that tailor content difficulty or feedback to individual learner progress are emerging in STEM education contexts (Crompton and Burke, 2023), with early applications in neuroscience training contexts.

However, the integration of AI features into DNRs introduces new dimensions of educational readiness that NERC does not yet explicitly capture. These include the transparency and explainability of AI-generated outputs, the risk of over-reliance that may undermine the development of critical scientific reasoning skills (Fan et al., 2025; Abrams et al., 2026), data privacy implications of AI services processing student interactions, and the potential for AI-generated content to introduce errors or biases in domain-specific contexts, for instance, hallucinated code in spike sorting workflows or inaccurate interpretations of neural data formats. Furthermore, the rapid pace of AI development raises sustainability concerns: features dependent on external AI services may change or become unavailable, affecting the long-term reliability of DNRs for educational use. Future NERC iterations should therefore incorporate AI-specific readiness criteria that evaluate not only whether AI features are present, but whether they are pedagogically aligned, transparent, and sustainable in educational settings. Technical readiness and infrastructure requirements, addressed in the third NERC domain, represent additional critical elements of educational equity and scalability. Infrastructure limitations, particularly when coupled with high costs, remain a primary driver of educational inequality (Barikzai et al., 2024). Such constraints should be critically evaluated during the development and refinement of DNRs. Assessment data consistently identify infrastructure barriers and high costs as significant hurdles to scalability. This suggests that economic viability functions as an implicit assessment criterion for medical and graduate schools. Future development strategies must therefore prioritize scalable, cost-effective solutions and open science tools to support equitable access to high-quality neuroscience education (Toofaninejad et al., 2025). Taken together, these findings suggest that improving neuroscience education is not solely a matter of developing better tools, but of designing tools that acknowledge the realities of teaching practice. By foregrounding educator readiness, NERC shifts attention from technological possibility to pedagogical feasibility.

## Validation approach and future psychometric evaluation

While the current expert-driven co-design process establishes content validity, systematic psychometric evaluation is essential to demonstrate NERC's predictive utility. Planned future efforts include longitudinal analyses to examine whether higher NERC scores correlate with real-world adoption metrics, such as instructor uptake and sustained classroom integration. A systematic evaluation of inter-rater reliability will further strengthen the checklist and sensitivity testing will evaluate NERC's capacity to detect meaningful improvements in educational readiness following iterative revisions informed by the checklist. By framing NERC as a formative reflection tool, we emphasize its role in making educator-facing barriers explicit early in the design process to improve eventual adoption outcomes.

## Future outlook and refinements of NERC

The field of educational technology is constantly evolving; and similarly, accessibility standards and requirements continue to evolve, requiring ongoing attention to ensure inclusivity. As a result, the criteria underlying NERC will require periodic refinement to remain aligned with emerging technologies, regulatory frameworks, and inclusive design principles. One persistent barrier lies in ensuring that DNR developers have sufficient resources (financial and time) to advance their DNR development to a certain level of education readiness suitable for classroom deployment. Continued dialogue between DNR developers, educators, and researchers will be essential to ensure that DNRs effectively meet the evolving needs of neuroscience teaching and learning requirements. While the current version of NERC represents an initial general framework, future work may include the development of more specialized versions tailored to specific educational levels, subdomains of neuroscience, or instructional modalities.

## Data Availability

The original contributions presented in the study are included in the article/Supplementary material, further inquiries can be directed to the corresponding author.
