# Supplementary material for: The neuroscience education readiness checklist for digital tools and methods
Source: Front Comput Neurosci. 2026 Jun 29;20:1812915. doi: 10.3389/fncom.2026.1812915 (PMC13357517; doi:10.3389/fncom.2026.1812915)
Supplement: Supplementary file 1 [file Data_Sheet_1.pdf]

Supplementary File: Annotated NERC Checklist

This annex provides an annotated version of the NERC Checklist. It complements the core checklist by providing interpretive guidance for those items using a traffic light evaluation logic, helping users apply the indicators consistently across diverse educational and institutional contexts.

The traffic-light evaluation logic (green, yellow, orange) used for the majority of items supports varying degrees of readiness and intends to capture the practical feasibility of deploying a DNR in real teaching settings.

Overall logic:

- Green: A “green” rating indicates that an item is largely met or poses minimal barriers to adoption for teaching purposes. Relevant information is fully provided.
- Yellow: A “yellow” rating signals conditional usability. Adoption for teaching may require additional effort, workarounds, support, or contextual knowledge from the instructor.
- Orange: An “orange” rating highlights substantial barriers. This could include high effort to ensure effective educational use, technical risks as well as unclear value or missing educational framing.

1. General items & accessibility

1e	Coding Competence Level	Beginner - Intermediate - Professional (mark the lowest entry barrier)	NA			

This item refers to the lowest coding competence level required for effective educational use by the intended learner group (not to optional advanced features).

Green: “Beginner or no coding skills required”: The DNR can be used without prior programming experience, or with only minimal exposure to coding concepts. All necessary code elements are pre-configured or clearly guided, allowing learners to focus on neuroscience rather than programming concepts.

Yellow: “Intermediate”: Effective use requires learners to write, modify or understand code (e.g. adapting scripts, …). However, the DNR provides sufficient pedagogical support, such as commented examples, step-by-step tutorials, templates, or troubleshooting guidance, so the resource is usable within a course setting.

Orange: “professional”: Use of this DNR requires advanced programming expertise and is not realistically usable by typical learners without substantial prior knowledge or training.

Pedagogical support is limited or absent, placing the learning focus on coding rather than on the intended neuroscience learning outcomes.

1f	Self-study	What is the level of self (or group) study involved? <33% (orange), Intermediate (yellow), >66% (green). (Mark the highest possible percentage)				

This item assesses the extent to which the DNR can be meaningfully used in self-paced or small-group learning settings, with limited reliance on continuous instructor presence.

Green: > 66 % self-study: The majority of the learning process using the DNR can be completed individually or in peer groups, as it is supported by clear instructions, structured learning paths and additional guidance (e.g. examples, tutorials). Learners can progress at their own pace, instructor input is mainly required for context or in-depth discussions.

Yellow: > 33 % self-study: A substantial portion of the DNR can be used independently, self-study feasible, but learning effects benefit from interaction with the instructor and some learning steps require synchronous instruction or facilitation.

Orange: <33 % self-study: The DNR relies heavily on real-time instructions, demonstration or live interactions with course instructors. Independent learning is difficult or impractical making the DNR difficult to integrate in asynchronous or blended learning formats without significant adaptation by the instructor.

1g	Clear & measurable education use cases	Are the educational use cases for this training resource clearly described? Are they specific to neuroscience topics, measurable, student-centric?	NA			

This item assesses to what extent the DNR is accompanied by explicitly articulated educational use cases that clarify what learners are expected to learn, how the DNR can be used in this context, and how learning outcomes can be observed or assessed.

Green: ‘Clear, neuroscience-specific, and measurable use cases’: Educational use cases are explicitly described and grounded in concrete neuroscience topics. They define student-centered learning objectives, specify the intended learner actions, and indicate observable and measurable outcomes (e.g. tasks completed, skills acquired).

Yellow: ‘General or partially specified educational use cases’: Educational use cases are described, but remain broad, tool-focused, or only loosely connected to neuroscience learning objectives. While potential educational value is apparent, learning goals or outcomes are not fully specified or need interpretation by the instructor.

Orange: ‘No clear educational use case’: The DNR lacks explicit educational use cases or presents primarily technical, demonstration- or research-oriented descriptions. Learning

objectives are either not defined, only implicit or developer-centric, making it difficult to assess how the resource supports student learning in a neuroscience education context.

1h	Accessible Multimedia	Visual information in multimedia such as videos or figures is described audibly or via text (captions/transcripts). All audio content includes transcripts.	NA			

This item assesses whether any multimedia content is designed to be accessible to learners with diverse needs, in line with inclusive educational principles.

Green: 'Fully accessible multimedia': All visual information (e.g. figures, videos, animations) is accompanied by textual or auditory descriptions. Videos include accurate captions and/or transcriptions, audio-only content is fully transcribed. Accessibility features are integrated as part of the standard learning materials without requiring additional effort from instructors.

Yellow: 'Partially accessible multimedia': Some accessibility features are provided, but coverage is incomplete or inconsistent. While the resource can be used by many learners, full accessibility across all material is not ensured.

Orange: 'Limited or no accessible multimedia': Captions, transcripts, or descriptive text are absent or minimal, creating significant barriers for learners with accessibility needs and limiting inclusive educational use.

1i	Active Engagement Strategy	Does it utilize interactivity, relevant multimedia, real-world neuroscience examples, collaboration, choice, or appropriate gamification to maintain student engagement?	NA			

This item examines whether the DNR actively incorporates pedagogical strategies to engage learners beyond passive content consumption.

Green: 'Active and intentional engagement strategies embedded in the resource': The DNR integrates structured opportunities for active learning such as interactive elements, authentic neuroscience examples, collaborative tasks or reflective / transfer-oriented exercises. Engagement activities are clearly aligned with learning objectives and support active student involvement throughout the learning process.

Yellow: 'Limited or instructor-dependent engagement': The DNR allows for active engagement, but interactive or engaging elements are rare, optional, or not pedagogically integrated. Meaningful engagement depends on additional facilitation by the instructor rather than being embedded in the resource itself.

Orange: 'Mostly passive learning experience': The resource relies mainly on passive modes of delivery with little opportunity for interaction, collaboration or active learners' engagement.

2. Instructor Usability & Workflow

2a	Setup Time & Complexity	How long is the initial setup time for the instructor and the course? Is it straightforward and efficient?	NA			

Green: 'Low setup effort and streamlined access': Setup is straightforward and can be completed with minimal preparation time. No individual account creation is required, or access is handled seamlessly via institutional systems (e.g. SSO). Instructors do not need to manually manage student accounts or permissions, and the DNR can be introduced within one teaching session using clear onboarding and setup guidance.

Yellow: 'Moderate setup effort with manageable complexity': Setup requires prior preparation and may involve individual user accounts, invitations or basic configuration. Self-registration is available, but instructors may need to coordinate access or troubleshoot in case of issues. Setup is feasible in standard teaching contexts, but setup time and complexity must be considered in course planning.

Orange: 'High setup effort and complex access management': Setup involves substantial technical or administrative effort, such as manual account creation, complex permission structures, or reliance on external support. Access management is unclear, and setup time is disproportionate to teaching time, creating a significant barrier to use in classroom settings.

2b	Classroom Management Features	Does it include relevant features for managing the online learning environment (e.g., communication, group management) if applicable?	NA			

This item assesses whether the DNR provides features that support instructors in managing learners and learning activities in an online or blended teaching setting, where applicable.

Green: 'Integrated classroom management support': The resource includes built-in features that support core classroom management needs, such as communication with learners, group or cohort management or role-based access.

Yellow: 'Limited or partially external classroom management': Some classroom management functions are available, but coverage is incomplete or relies on external systems (e.g. separate communication channels). Classroom management remains manageable, but instructors must coordinate across multiple tools to support the overall learning environment.

Orange: 'No classroom management support': The resource provides no features for managing learners, communication, or group activities. Instructors must rely entirely on external platforms to organize and support the learning process.

2c	Clear & Accessible Documentation	Does it provide comprehensive, well-organized, and easily searchable user manuals, FAQs, and technical documentation for instructors?	NA			

This items evaluates the availability and quality of instructor-oriented documentation that supports adoption, troubleshooting and sustainable educational use of the DNR.

Green: 'Comprehensive and instructor-friendly documentation': The resource provides well-structured, up-to-date and easily searchable documentation tailored to instructional use. This includes clear user and instructor manuals, FAQs, tutorials or technical guidance that specifically address common teaching scenarios and problems.

Yellow: 'Partial or technically focused documentation': Documentation is available but may be either incomplete or primarily oriented towards technical or developer audiences. Key information for instructors exists but may not be easy to find, interpret or adapt to educational contexts. Additional support or prior experience of the instructor may be needed.

Orange: 'Insufficient or inaccessible documentation': Documentation is sparse, outdated or difficult to navigate. Instructor-relevant guidance is missing, making effective educational use dependent on direct support from developers or trial-and-error approaches.

2d	Availability of instructor packages	Does it come with existing free-to-use instructor packages, such as presentations, exercises or use cases that can be easily re-utilized in classrooms?	NA			

This item assesses whether the DNR is accompanied by ready-to-use educational materials that reduce preparation effort for instructors.

Green: 'Comprehensive, reusable instructor packages': The DNR includes freely available instructor packages such as presentations, exercises, worksheets or clearly defined educational use cases that can be easily reused or adapted for classroom teaching. Materials are pedagogically structured, aligned with learning objectives, and suitable for integration in existing courses.

Yellow: 'Limited or partially reusable materials': Some instructor-oriented materials are provided, but they may be incomplete, loosely structured or need substantial adaptations

before use. Materials may be helpful as a starting point, but instructors must invest additional efforts to contextualize or redesign materials to meet the teaching needs.

Orange: 'No instructor packages available': The resource does not provide any dedicated instructor materials. Instructors need to develop all teaching content independently.

2e	Efficient Student Progress Monitoring	Does the tool provide clear, actionable data/possibilities for instructors to easily track student engagement, progress, and performance?	NA			

This item examines whether the resource provides meaningful mechanisms for instructors to monitor student engagement, learning progress and performance.

Green: 'Clear and actionable progress monitoring': The tool offers built-in features that allow instructors to easily track student activity, progress or completion (e.g. dashboards, logs, summaries, or exportable data). Information is presented in a clear and interpretable manner and supports feedback or assessment without the need for additional technical processing.

Yellow: 'Limited or indirect progress monitoring': Some information on student engagement or progress is available, but may require additional technical steps or manual interpretation (e.g. raw logs, indirect progress indicators). Monitoring is feasible but additional effort or technical competence from the instructor is required.

Orange: 'No progress monitoring': The resources do not provide usable means for instructors to monitor student learning progress or performance.

2f	Facilitation of Peer Support / Community	Does it provide mechanisms (e.g., forums) for instructors using the resource to connect and share practices?	NA			

This item assesses whether the resource supports exchange, collaboration and peer learning among instructors.

Green: 'Active and accessible instructor community': The resource provides established mechanisms for peer exchange, such as forums, discussion spaces, mailing lists or community platforms that are actively used by instructors to share teaching practices, use cases, challenges and solutions.

Yellow: 'Informal or limited peer support': Opportunities for peer exchange exist but are informal, sparsely used, or external to the resource (e.g. ad hoc channels, occasional

workshops). While peer support is possible, it is not systematically facilitated or easily findable for new instructors.

Orange: 'No peer support or community mechanism': The resource does not offer any structured means for instructors to connect, exchange experiences or share educational practices.

2g	Instructor Flexibility & Customization Options	Does it allow instructors some control to adapt the resource (e.g., select content, adjust settings, add context) to fit specific course needs and teaching styles?	NA			

This item examines the extent to which instructors can adapt the resource to their specific teaching context, learning objectives, and pedagogical approach.

Green: 'High flexibility and meaningful customization': The resource allows instructors to easily adapt content, structure or settings (e.g. selecting or ordering materials, adjusting parameters, adding context information, integrating own examples). Customization does not require technical workarounds.

Yellow: 'Limited or constrained customization': Instructors can tailor the resource to some extent., but flexibility is restricted to predefined options or requires additional effort (e.g. manual adjustments, external tools or reconfigurations).

Orange: 'Minimal or no customization options': The resource enforces a fixed structure or workflow that cannot be meaningfully adapted to different teaching contexts. Instructors have little to no control over content, sequence or presentation.

2h	Guidance on Assessment	Does it provide specific guidance, resources, or frameworks for effective assessment/grading?	NA			

This item assesses whether the resource provides explicit support for assessing student learning, including guidance on assessment and grading strategies.

Green: 'Clear and actionable assessment guidance': The resource includes concrete recommendations, examples or frameworks for assessing student learning (e.g. suggested assessment tasks, criteria, or alignment with learning objectives).

Yellow: 'Implicit or partial assessment guidance': Assessment is implied through activities or outputs, but explicit guidance on how to evaluate student performance is limited. Instructors

must interpret or design assessments themselves, using the resources primarily as a learning activity rather than an assessable component.

Orange: ‘No or minimal assessment guidance provided’: The resource does not address assessment considerations and does not offer support for evaluating student learning.

3. Technical Readiness

3a	Installation requirements	Does the DNR need installation? If **Yes**, is it platform independent? Does it need soft-or specific hardware prerequisites? Does it require software dependencies prone to regular updates (high risk of issues)?	NA			

This item assesses the technical effort and risk associated with installing and maintaining the resource for educational use including platform dependence, software dependencies and hardware requirements.

Green: ‘Minimal or no installation requirements’: The resource is browser-based or requires only minimal installation that is platform-independent and stable. No specialized hardware is required, and software dependencies are limited, well-managed and unlikely to cause compatibility issues. Installation does not require administrative privileges or institutional IT interventions.

Yellow: ‘Manageable installation with some dependencies’: Installation is required and may involve specific software dependencies, operating systems, or moderate hardware requirements. While feasible in standard teaching environments, setup may require advance preparation, testing, or support from institutional IT services. Ongoing maintenance (e.g. updates) must be anticipated.

Orange: ‘Complex or fragile installation requirements’: Installation is technically complex, platform-specific or dependent on rapidly changing software environments. Specialized hardware or frequent manual updates are required, and incompatibility issues are likely.

3b	Minimal & Transparent Technical Requirements	What are the technical requirements? Does it minimize the need for specialized hardware/software or high-speed internet? Does it clearly communicate requirements? Does it consider equity? Does it avoid requiring users to download and install additional software or browser plug-ins where possible?	NA			

This item assesses whether the resource defines clear, realistic and equitable technical requirements for educational use, and whether these requirements are transparently communicated to instructors and learners.

Green: ‘Minimal, transparent, and equitable requirements’: Technical requirements are clearly documented, easy to understand, and compatible with standard institutional infrastructure and commonly available devices. The resource avoids specialized hardware, proprietary software, or high-bandwidth dependencies where possible. It does not require additional downloads or browser-plugins beyond standard environments. Equity and access considerations are explicitly or implicitly supported.

Yellow: ‘Moderate or partially transparent requirements’: Technical requirements are reasonable but include some constraints (e.g. higher bandwidth, specific software versions, or limited device compatibility). Requirements are documented but may lack clarity or be distributed across sources. While most learners can access the resource, some may require institutional support or workarounds.

Orange: ‘High or unclear technical requirements’: Technical requirements are poorly documented or difficult to meet in typical teaching contexts. The resource relies on specialized hardware, restrictive software environments, or high-performance computing and connectivity without clearly communicating prerequisites.

3c	Reliability & Stability	Does the resource function consistently without frequent crashes, errors, or bugs on standard platforms/OS.	NA			

This item examines whether the resource functions consistently and predictably under typical educational conditions, without disrupting teaching and learning activities.

Green: ‘High reliability and stable performance’: The resource operatives reliably on standard platforms and operating systems with minimal downtime, crashes or errors during regular use. Performance is stable under expected classroom concurrent loads, and known issues are rare, well-documented, or promptly resolved.

Yellow: ‘Occasional or context-dependent reliability issues’: The resource is generally functional but may exhibit occasional errors, performance slowdowns, or compatibility issues under certain conditions (e.g. peak usage, specific devices). While teaching remains feasible, instructors should anticipate potential disruptions and have contingency plans.

Orange: ‘Frequent instability or unreliably performance’: The resource is prone to crashes, errors, or unpredictable behaviour that regularly interrupts teaching activities. Reliability issues

are common or unresolved, making the resource unsuitable for sustained or time-critical educational use.

3d	Transparent & Secure Data Privacy Practices	Adheres to relevant privacy laws (FERPA, GDPR); clearly communicates data collection, usage, storage, security, and sharing policies. Minimizes PII collection. TrustEd Apps vetting is a plus.	NA			

This item evaluates whether the resource adheres to relevant data protection and privacy standards and transparently communicates its data handling practices in an educational context.

Green: 'Clear, compliant, and transparent data privacy practices': The resource complies with applicable data protection regulations (e.g. GDPR, FERPA), and clearly communicates what data is collected, how it is stored, used and protected, and whether it is shared with third parties. Collection of personally identifiable information (PII) is minimized, and institutional or TrustED Apps vetting (if applicable) supports confidence in educational use.

Yellow: 'Partial or unclear privacy transparency': Data privacy information is available, but incomplete, difficult to interpret, or lacks specificity regarding educational use. While no obvious violations are evident, instructors or institutions may need to conduct additional checks or define constraints before adoption.

Orange: 'Insufficient or non-compliant privacy practices': Data privacy policies are absent, insufficient or indicate non-compliance with relevant regulations. Data handling practices are unclear or inappropriate for educational contexts, creating significant legal, ethical or institutional barriers to use.

3e	Clear Data Ownership & Portability	Do policies clearly state ownership of data, reuse, and clarify ownership of user-generated content? Does the resource provide mechanisms for users to export/archive their data?	NA			

This item assesses whether the resource provides transparent policies on data ownership and practical mechanisms for data re-use and portability, which are essential for sustainable educational use.

Green: 'Clear ownership and easy data portability': Data ownership is explicitly stated, including ownership of user-generated content and learning artefacts. Policies clearly define rights for

reuse and retention, and the resource provides straightforward mechanisms for exporting or archiving data in standard formats.

Yellow: 'Partial clarity on ownership or limited portability': Data ownership is addressed but lacks detail or clarity in specific scenarios (e.g. reuse of student-generated content). Data export is possible but limited in scope, or usability. Portability is manageable, but instructors or institutions may need to apply additional mechanisms or workarounds.

Orange: 'Unclear ownership or no data portability': Data ownership is ambiguous or undocumented, and users have no practical means to export, archive or reuse their data.

4. Interpreting NERC Results

NERC is a framework designed as a formative reflection tool, where individual items are not weighted but the pattern of ratings across the three domains provides a qualitative profile of a DNR's education readiness, highlighting specific areas of strength or requiring attention.

- A DNR with predominantly green ratings and isolated yellow or orange items may be considered broadly ready for educational deployment.
- A concentration of orange items within a single domain may indicate systemic barriers to adoption that should be addressed before classroom deployment. Users are encouraged to prioritize items rated orange, as these typically represent the most consequential barriers to effective use.
- Items marked NA should be excluded from the overall assessment profile rather than treated as deficiencies. A high proportion of NAs may indicate that the checklist is being applied outside its intended scope, and NERC might not be the appropiate evaluation tool for the particular resource.
- NERC is most effective when the results are used for improvement, iteratively. Users are encouraged to start a first assessment, address identified barriers, and re-evaluate using the checklist to track progress over time. Sharing completed checklists with educators or users can also provide valuable external validation.

Supplementary Table 1: Summary of NERC color-coded distributions across the three domains for the five example DNRs.

This table provides a structured, at-a-glance qualitative comparison of educational readiness, enabling direct visual evaluation of relative strengths and improvement areas between the example DNRs.

DNR	GENERAL ITEMS & ACCESSIBILITY	INSTRUCTOR USABILITY & WORKFLOW	TECHNICAL READINESS	OVERALL PATTERN
**ADVANCED COMPUTATIONAL MODELING MODULE**	3 Green / 2 Yellow / 0 Orange	4 Green / 4 Yellow / 0 Orange	4 Green / 1 Yellow / 0 Orange	Relatively high scoring throughout, mixed in Domain 2
**NIH TOOLBOX**	2 Green / 2 Yellow / 0 Orange / 1 NA	3 Green / 3 Yellow / 2 Orange / 0 NA	5 Green / 0 Yellow / 0 Orange / 0 NA	Strong in Domain 3, mixed in domains 1 and 2
**MESHVIEW**	4 Green / 1 Yellow / 0 Orange / 0 NA	2 Green / 1 Yellow / 5 Orange / 0 NA	4 Green / 0 Yellow / 0 Orange 1 NA	Strong in Domains 1 & 3, weak in 2
**ELEPHANT**	1 Green / 1 Yellow / 3 Orange / 0 NA	2 Green / 2 Yellow / 1 Orange / 3 NA	4 Green / 0 Yellow / 0 Orange / 1 NA	Strongest in Domain 3
**BRAINSCALES-2**	3 Green / 2 Yellow / 0 Orange/ 0 NA	1 Green / 5 Yellow / 2 Orange/ 0 NA	4 Green / 1 Yellow / 0 Orange/ 0 NA	Strong in accessibility and technical readiness; needs improvement in instructor domain

Note: Green = high readiness / Orange = substantial barriers / Yellow = conditional usability.

Supplementary Table 2: NERC Checklist for Example 1: Advanced Computational Modeling and Inference Training Module

Item Nr.	Checklist Criteria	Description	Self Evaluation			
		**1. General Items & Accessibility**				
1a	Usage	Number of students trained so far (estimate)	> 100 in the previous 2-3 years			
1b	Language Availability and requirement	In which languages is the DNR available? (e.g. English)	English			
1c	Associated Cost Estimate	What are the direct costs associated with the training resource for the trainer? (e.g. license cost)	no cost			
1d	Educational Level	What is the expected education level target group? (Mark several if applicable)	Primary			
			Secondary			
			Undergraduate			
			Graduate			x
			Post-Graduate			x
1e	Coding Competence Level	Beginner (green) – Intermediate (yellow) - Professional (orange) (mark the lowest entry barrier)	NA			x
1f	Self-study	What is the level of self (or group) study involved? <33% (orange), Intermediate (yellow), >66% (green). (Mark the highest possible percentage)				x
1g	Clear & measurable education use cases	Are the educational use cases for this training resource clearly described? Are they specific to neuroscience topics, measurable, student-centric? What are the Intended Learning Outcomes?	NA			x
1h	Accessible Multimedia	Visual information in multimedia such as videos or figures is described audibly or via text (captions/transcripts). All audio content includes transcripts.	NA		x	
1i	Active Engagement Strategy	Does it utilize interactivity, relevant multimedia, real-world neuroscience examples, collaboration, choice, or appropriate gamification to maintain student engagement?	NA		x	
		**2. Instructor Usability & Workflow**				
2a	Setup Time & Complexity	How long is the initial setup time for the instructor and the course? Is it straightforward and efficient?	NA			x
2b	Classroom Management Features	Does it include relevant features for managing the online learning environment (e.g., communication, group management) if applicable?	NA			x
2c	Clear & Accessible Documentation	Does it provide comprehensive, well-organized, and easily searchable user manuals, FAQs, and technical documentation for instructors?	NA			x
2d	Availability of instructor packages	Does it come with existing free-to-use instructor packages, such as presentations, exercises or use cases that can be easily re-utilized in classrooms?	NA			x
2e	Efficient Student Progress Monitoring	Does the tool provide clear, actionable data/possibilities for instructors to easily track student engagement, progress, and performance?	NA		x	
2f	Facilitation of Peer Support / Community	Does it provide mechanisms (e.g., forums) for instructors using the resource to connect and share practices?	NA		x	
2g	Instructor Flexibility & Customization Options	Does it allow instructors some control to adapt the resource (e.g., select content, adjust settings, add context) to fit specific course needs and teaching styles?	NA		x	
2h	Guidance on Assessment	Does it provide specific guidance, resources, or frameworks for effective assessment/grading?	NA		x	
		**3. Technical Readiness**				
3a	Installation requirements	Does the DNR need installation? If Yes, is it platform independent? Does it need soft-or specific hardware prerequisites? Does it require software dependencies prone to regular updates (high risk of issues)?	NA			x
3b	Minimal & Transparent Technical Requirements	What are the technical requirements? Does it minimize the need for specialized hardware/software or high-speed internet? Does it clearly communicate requirements? Does it consider equity? Does it avoid requiring users to download and install additional software or browser plug-ins where possible?	NA			x
3c	Reliability & Stability	Does the resource function consistently without frequent crashes, errors, or bugs on standard platforms/OS.	NA			x
3d	Transparent & Secure Data Privacy Practices	Adheres to relevant privacy laws (FERPA, GDPR); clearly communicates data collection, usage, storage, security, and sharing policies. Minimizes PII collection. TrustEd Apps vetting is a plus.	NA			x
3e	Clear Data Ownership & Portability	Do policies clearly state ownership of data, reuse, and clarify ownership of user-generated content? Does the resource provide mechanisms for users to export/archive their data?	NA		x	
3f	Scalability	Can the infrastructure handle expected user loads (varying class sizes, concurrent use) without performance issues? What is the limit? (state number of users)	NA	The tool supports parallelisation and can scale to larger groups. Capacity depends on the available infrastructure, such as the number of EBRAINS nodes. Max. concurrent users in previous courses: 30.		
3g	Offline Capability	Can core functionality operate without continuous internet access? Critical for field-based teaching or institutions with limited connectivity.		On-line	**Partial**<br>x	Full
3h	LTI /Learning Tools Interoperability) Conformance	Supports LTI standards.	NA			

Supplementary Table 3: NERC Checklist for Example 2: NIH Toolbox - Standardized Neurobehavioral Assessment Platform

Item Nr.	Checklist Criteria	Description	Self Evaluation			
**1. General Items & Accessibility**						
1a	Usage	Number of students trained so far (estimate)	estimated 10,000			
1b	Language Availability and requirement	In which languages is the DNR available? (e.g. English)	English, Spanish, French, Arabic, Hebrew, Italian			
1c	Associated Cost Estimate	What are the direct costs associated with the training resource for the trainer? (e.g. license cost)	Downloadable from the Apple App Store: Free<br><br>Subscription cost for test scores and data storage:<br>12-month subscription for $499.99<br><br>Hardware costs for equipment or material needed to use the toolbox: Cost varies as described here: https://www.healthmeasures.net/images/nihtoolbox/Training-Admin-Scoring_Manuals/NIH_Toolbox_App_Administrators_Manual_v1.17.pdf			
1d	Educational Level	What is the expected education level target group? (Mark several if applicable)	Primary			x
			Secondary			x
			Undergraduate			x
			Graduate			x
			Post-Graduate			x
1e	Coding Competence Level	Beginner (green) – Intermediate (yellow) - Professional (orange) (mark the lowest entry barrier)	**NA**			
1f	Self-study	What is the level of self (or group) study involved? <33% (orange), Intermediate (yellow), >66% (green). (Mark the highest possible percentage)				x
1g	Clear & measurable education use cases	Are the educational use cases for this training resource clearly described? Are they specific to neuroscience topics, measurable, student-centric? What are the Intended Learning Outcomes?	NA			x
1h	Accessible Multimedia	Visual information in multimedia such as videos or figures is described audibly or via text (captions/transcripts). All audio content includes transcripts.	NA		x	
1i	Active Engagement Strategy	Does it utilize interactivity, relevant multimedia, real-world neuroscience examples, collaboration, choice, or appropriate gamification to maintain student engagement?	NA		x	
**2. Instructor Usability & Workflow**						
2a	Setup Time & Complexity	How long is the initial setup time for the instructor and the course? Is it straightforward and efficient?	NA			x
2b	Classroom Management Features	Does it include relevant features for managing the online learning environment (e.g., communication, group management) if applicable?	NA		x	
2c	Clear & Accessible Documentation	Does it provide comprehensive, well-organized, and easily searchable user manuals, FAQs, and technical documentation for instructors?	NA			x
2d	Availability of instructor packages	Does it come with existing free-to-use instructor packages, such as presentations, exercises or use cases that can be easily re-utilized in classrooms?	NA	x		
2e	Efficient Student Progress Monitoring	Does the tool provide clear, actionable data/possibilities for instructors to easily track student engagement, progress, and performance?	NA		x	
2f	Facilitation of Peer Support / Community	Does it provide mechanisms (e.g., forums) for instructors using the resource to connect and share practices?	NA	x		
2g	Instructor Flexibility & Customization Options	Does it allow instructors some control to adapt the resource (e.g., select content, adjust settings, add context) to fit specific course needs and teaching styles?	NA			x
2h	Guidance on Assessment	Does it provide specific guidance, resources, or frameworks for effective assessment/grading?	NA		x	
**3. Technical Readiness**						
3a	Installation requirements	Does the DNR need installation? If Yes, is it platform independent? Does it need soft-or specific hardware prerequisites? Does it require software dependencies prone to regular updates (high risk of issues)?	NA			x
3b	Minimal & Transparent Technical Requirements	What are the technical requirements? Does it minimize the need for specialized hardware/software or high-speed internet? Does it clearly communicate requirements? Does it consider equity? Does it avoid requiring users to download and install additional software or browser plug-ins where possible?	NA			x
3c	Reliability & Stability	Does the resource function consistently without frequent crashes, errors, or bugs on standard platforms/OS.	NA			x
3d	Transparent & Secure Data Privacy Practices	Adheres to relevant privacy laws (FERPA, GDPR); clearly communicates data collection, usage, storage, security, and sharing policies. Minimizes PII collection. TrustEd Apps vetting is a plus.	NA			x
3e	Clear Data Ownership & Portability	Do policies clearly state ownership of data, reuse, and clarify ownership of user-generated content? Does the resource provide mechanisms for users to export/archive their data?	NA			x
3f	Scalability	Can the infrastructure handle expected user loads (varying class sizes, concurrent use) without performance issues? What is the limit? (state number of users)	NA	yes, no strict limit		
3g	Offline Capability	Can core functionality operate without continuous internet access? Critical for field-based teaching or institutions with limited connectivity.		On-line	Partial	**Full**<br>x
3h	LTI /Learning Tools Interoperability) Conformance	Supports LTI standards.	**NA**			

Supplementary Table 4: NERC Checklist for Example 3: MeshView - 3D Atlas Viewer

Item Nr.	Checklist Criteria	Description	Self Evaluation			
**1. General Items & Accessibility**						
1a	Usage	Number of students trained so far (estimate)	> 250			
1b	Language Availability and requirement	In which languages is the DNR available? (e.g. English)	English			
1c	Associated Cost Estimate	What are the direct costs associated with the training resource for the trainer? (e.g. license cost)	None so far			
1d	Educational Level	What is the expected education level target group? (Mark several if applicable)	Primary			
			Secondary			
			Undergraduate			x
			Graduate			x
			Post-Graduate			x
1e	Coding Competence Level	Beginner (green) – Intermediate (yellow) - Professional (orange) (mark the lowest entry barrier)	NA			x
1f	Self-study	What is the level of self (or group) study involved? <33% (orange), Intermediate (yellow), >66% (green). (Mark the highest possible percentage)				x
1g	Clear & measurable education use cases	Are the educational use cases for this training resource clearly described? Are they specific to neuroscience topics, measurable, student-centric? What are the Intended Learning Outcomes?	NA		x	
1h	Accessible Multimedia	Visual information in multimedia such as videos or figures is described audibly or via text (captions/transcripts). All audio content includes transcripts.	NA			x
1i	Active Engagement Strategy	Does it utilize interactivity, relevant multimedia, real-world neuroscience examples, collaboration, choice, or appropriate gamification to maintain student engagement?	NA			x
**2. Instructor Usability & Workflow**						
2a	Setup Time & Complexity	How long is the initial setup time for the instructor and the course? Is it straightforward and efficient?	NA			x
2b	Classroom Management Features	Does it include relevant features for managing the online learning environment (e.g., communication, group management) if applicable?	NA	x		
2c	Clear & Accessible Documentation	Does it provide comprehensive, well-organized, and easily searchable user manuals, FAQs, and technical documentation for instructors?	NA			x
2d	Availability of instructor packages	Does it come with existing free-to-use instructor packages, such as presentations, exercises or use cases that can be easily re-utilized in classrooms?	NA	x		
2e	Efficient Student Progress Monitoring	Does the tool provide clear, actionable data/possibilities for instructors to easily track student engagement, progress, and performance?	NA	x		
2f	Facilitation of Peer Support / Community	Does it provide mechanisms (e.g., forums) for instructors using the resource to connect and share practices?	NA	x		
2g	Instructor Flexibility & Customization Options	Does it allow instructors some control to adapt the resource (e.g., select content, adjust settings, add context) to fit specific course needs and teaching styles?	NA	x		
2h	Guidance on Assessment	Does it provide specific guidance, resources, or frameworks for effective assessment/grading?	NA		x	
**3. Technical Readiness**						
3a	Installation requirements	Does the DNR need installation? If Yes, is it platform independent? Does it need soft-or specific hardware prerequisites? Does it require software dependencies prone to regular updates (high risk of issues)?	NA			x
3b	Minimal & Transparent Technical Requirements	What are the technical requirements? Does it minimize the need for specialized hardware/software or high-speed internet? Does it clearly communicate requirements? Does it consider equity? Does it avoid requiring users to download and install additional software or browser plug-ins where possible?	NA			x
3c	Reliability & Stability	Does the resource function consistently without frequent crashes, errors, or bugs on standard platforms/OS.	NA			x
3d	Transparent & Secure Data Privacy Practices	Adheres to relevant privacy laws (FERPA, GDPR); clearly communicates data collection, usage, storage, security, and sharing policies. Minimizes PII collection. TrustEd Apps vetting is a plus.	**NA**			
3e	Clear Data Ownership & Portability	Do policies clearly state ownership of data, reuse, and clarify ownership of user-generated content? Does the resource provide mechanisms for users to export/archive their data?	NA			x
3f	Scalability	Can the infrastructure handle expected user loads (varying class sizes, concurrent use) without performance issues? What is the limit? (state number of users)	NA	MeshView runs in the browser, with some data downloaded locally. Capacity depends on the underlying infrastructure and individual users' network connections. No user complaints have been recorded; previous courses supported up to 25 concurrent users.		
3g	Offline Capability	Can core functionality operate without continuous internet access? Critical for field-based teaching or institutions with limited connectivity.		**On-line** x	Partial	Full
3h	LTI /Learning Tools Interoperability) Conformance	Supports LTI standards.	NA	No but uses EBRAINS IAM aut system and connects to the infrastructure		

Supplementary Table 5: NERC Checklist for Example 4: Elephant Analysis Toolkit

Item Nr.	Checklist Criteria	Description	Self Evaluation			
**1. General Items & Accessibility**						
1a	Usage	Number of students trained so far (estimate)	500			
1b	Language Availability and requirement	In which languages is the DNR available? (e.g. English)	English			
1c	Associated Cost Estimate	What are the direct costs associated with the training resource for the trainer? (e.g. license cost)	No cost			
1d	Educational Level	What is the expected education level target group? (Mark several if applicable)	Primary			
			Secondary			
			Undergraduate			x
			Graduate			x
			Post-Graduate			x
1e	Coding Competence Level	Beginner (green) – Intermediate (yellow) - Professional (orange) (mark the lowest entry barrier)	NA		x	
1f	Self-study	What is the level of self (or group) study involved? <33% (orange), Intermediate (yellow), >66% (green). (Mark the highest possible percentage)				x
1g	Clear & measurable education use cases	Are the educational use cases for this training resource clearly described? Are they specific to neuroscience topics, measurable, student-centric? What are the Intended Learning Outcomes?	NA	x		
1h	Accessible Multimedia	Visual information in multimedia such as videos or figures is described audibly or via text (captions/transcripts). All audio content includes transcripts.	NA	x		
1i	Active Engagement Strategy	Does it utilize interactivity, relevant multimedia, real-world neuroscience examples, collaboration, choice, or appropriate gamification to maintain student engagement?	NA	x		
**2. Instructor Usability & Workflow**						
2a	Setup Time & Complexity	How long is the initial setup time for the instructor and the course? Is it straightforward and efficient?	NA			x
2b	Classroom Management Features	Does it include relevant features for managing the online learning environment (e.g., communication, group management) if applicable?	NA			
2c	Clear & Accessible Documentation	Does it provide comprehensive, well-organized, and easily searchable user manuals, FAQs, and technical documentation for instructors?	NA			x
2d	Availability of instructor packages	Does it come with existing free-to-use instructor packages, such as presentations, exercises or use cases that can be easily re-utilized in classrooms?	NA	x		
2e	Efficient Student Progress Monitoring	Does the tool provide clear, actionable data/possibilities for instructors to easily track student engagement, progress, and performance?	NA			
2f	Facilitation of Peer Support / Community	Does it provide mechanisms (e.g., forums) for instructors using the resource to connect and share practices?	NA		x	
2g	Instructor Flexibility & Customization Options	Does it allow instructors some control to adapt the resource (e.g., select content, adjust settings, add context) to fit specific course needs and teaching styles?	NA		x	
2h	Guidance on Assessment	Does it provide specific guidance, resources, or frameworks for effective assessment/grading?	NA			
**3. Technical Readiness**						
3a	Installation requirements	Does the DNR need installation? If Yes, is it platform independent? Does it need soft-or specific hardware prerequisites? Does it require software dependencies prone to regular updates (high risk of issues)?	NA			x
3b	Minimal & Transparent Technical Requirements	What are the technical requirements? Does it minimize the need for specialized hardware/software or high-speed internet? Does it clearly communicate requirements? Does it consider equity? Does it avoid requiring users to download and install additional software or browser plug-ins where possible?	NA			x
3c	Reliability & Stability	Does the resource function consistently without frequent crashes, errors, or bugs on standard platforms/OS.	NA			x
3d	Transparent & Secure Data Privacy Practices	Adheres to relevant privacy laws (FERPA, GDPR); clearly communicates data collection, usage, storage, security, and sharing policies. Minimizes PII collection. TrustEd Apps vetting is a plus.	NA			
3e	Clear Data Ownership & Portability	Do policies clearly state ownership of data, reuse, and clarify ownership of user-generated content? Does the resource provide mechanisms for users to export/archive their data?	NA			x
3f	Scalability	Can the infrastructure handle expected user loads (varying class sizes, concurrent use) without performance issues? What is the limit? (state number of users)	NA			
3g	Offline Capability	Can core functionality operate without continuous internet access? Critical for field-based teaching or institutions with limited connectivity.		On-line	Partial	**Full** x
3h	LTI /Learning Tools Interoperability) Conformance	Supports LTI standards.	NA	no		

Supplementary Table 6: NERC Checklist for Example 5: BrainScaleS-2 - Neuromorphic System

Item Nr.	Checklist Criteria	Description	Self Evaluation			
**1. General Items & Accessibility**						
1a	Usage	Number of students trained so far (estimate)	approx. 1,000			
1b	Language Availability and requirement	In which languages is the DNR available? (e.g. English)	English/German (easily machine translatable)			
1c	Associated Cost Estimate	What are the direct costs associated with the training resource for the trainer? (e.g. license cost)	no cost			
1d	Educational Level	What is the expected education level target group? (Mark several if applicable)	Primary			
			Secondary			x
			Undergraduate			x
			Graduate			x
			Post-Graduate			x
1e	Coding Competence Level	Beginner (green) – Intermediate (yellow) - Professional (orange) (mark the lowest entry barrier)	NA			x
1f	Self-study	What is the level of self (or group) study involved? <33% (orange), Intermediate (yellow), >66% (green). (Mark the highest possible percentage)				x
1g	Clear & measurable education use cases	Are the educational use cases for this training resource clearly described? Are they specific to neuroscience topics, measurable, student-centric? What are the Intended Learning Outcomes?	NA		x	
1h	Accessible Multimedia	Visual information in multimedia such as videos or figures is described audibly or via text (captions/transcripts). All audio content includes transcripts.	NA		x	
1i	Active Engagement Strategy	Does it utilize interactivity, relevant multimedia, real-world neuroscience examples, collaboration, choice, or appropriate gamification to maintain student engagement?	NA			x
**2. Instructor Usability & Workflow**						
2a	Setup Time & Complexity	How long is the initial setup time for the instructor and the course? Is it straightforward and efficient?	NA		x	
2b	Classroom Management Features	Does it include relevant features for managing the online learning environment (e.g., communication, group management) if applicable?	NA	x		
2c	Clear & Accessible Documentation	Does it provide comprehensive, well-organized, and easily searchable user manuals, FAQs, and technical documentation for instructors?	NA		x	
2d	Availability of instructor packages	Does it come with existing free-to-use instructor packages, such as presentations, exercises or use cases that can be easily re-utilized in classrooms?	NA		x	
2e	Efficient Student Progress Monitoring	Does the tool provide clear, actionable data/possibilities for instructors to easily track student engagement, progress, and performance?	NA	x		
2f	Facilitation of Peer Support / Community	Does it provide mechanisms (e.g., forums) for instructors using the resource to connect and share practices?	NA		x	
2g	Instructor Flexibility & Customization Options	Does it allow instructors some control to adapt the resource (e.g., select content, adjust settings, add context) to fit specific course needs and teaching styles?	NA			x
2h	Guidance on Assessment	Does it provide specific guidance, resources, or frameworks for effective assessment/grading?	NA		x	
**3. Technical Readiness**						
3a	Installation requirements	Does the DNR need installation? If Yes, is it platform independent? Does it need soft-or specific hardware prerequisites? Does it require software dependencies prone to regular updates (high risk of issues)?	NA			x
3b	Minimal & Transparent Technical Requirements	What are the technical requirements? Does it minimize the need for specialized hardware/software or high-speed internet? Does it clearly communicate requirements? Does it consider equity? Does it avoid requiring users to download and install additional software or browser plug-ins where possible?	NA			x
3c	Reliability & Stability	Does the resource function consistently without frequent crashes, errors, or bugs on standard platforms/OS.	NA		x	
3d	Transparent & Secure Data Privacy Practices	Adheres to relevant privacy laws (FERPA, GDPR); clearly communicates data collection, usage, storage, security, and sharing policies. Minimizes PII collection. TrustEd Apps vetting is a plus.	NA			x
3e	Clear Data Ownership & Portability	Do policies clearly state ownership of data, reuse, and clarify ownership of user-generated content? Does the resource provide mechanisms for users to export/archive their data?	NA			x
3f	Scalability	Can the infrastructure handle expected user loads (varying class sizes, concurrent use) without performance issues? What is the limit? (state number of users)	NA	around 40		
3g	Offline Capability	Can core functionality operate without continuous internet access? Critical for field-based teaching or institutions with limited connectivity.		**On-line** x	Partial	Full
3h	LTI /Learning Tools Interoperability) Conformance	Supports LTI standards.	NA	no		
